# Feeding and Stocking Up: Radio-Labelled Food Reveals Exchange Patterns in Ants

**DOI:** 10.1371/journal.pone.0005919

**Published:** 2009-06-17

**Authors:** Aurélie Buffin, Damien Denis, Gaetan Van Simaeys, Serge Goldman, Jean-Louis Deneubourg

**Affiliations:** 1 Unit of Social Ecology, Université libre de Bruxelles, Brussels, Belgium; 2 Hôpital Erasme Service of Nuclear Medicine, Université libre de Bruxelles, Brussels, Belgium; University of Bristol, United Kingdom

## Abstract

Food sharing is vital for a large number of species, either solitary or social, and is of particular importance within highly integrated societies, such as in colonial organisms and in social insects. Nevertheless, the mechanisms that govern the distribution of food inside a complex organizational system remain unknown.

Using scintigraphy, a method developed for medical imaging, we were able to describe the dynamics of food-flow inside an ant colony. We monitored the sharing process of a radio-labelled sucrose solution inside a nest of *Formica fusca*. Our results show that, from the very first load that enters the nest, food present within the colony acts as negative feedback to entering food. After one hour of the experiments, 70% of the final harvest has already entered the nest. The total foraged quantity is almost four times smaller than the expected storage capacity. A finer study of the spatial distribution of food shows that although all ants have been fed rapidly (within 30 minutes), a small area representing on average 8% of the radioactive surface holds more than 25% of the stored food. Even in rather homogeneous nests, we observed a strong concentration of food in few workers. Examining the position of these workers inside the nest, we found heavily loaded ants in the centre of the aggregate. The position of the centre of this high-intensity radioactive surface remained stable for the three consecutive hours of the experiments.

We demonstrate that the colony simultaneously managed to rapidly feed all workers (200 ants fed within 30 minutes) and build up food stocks to prevent food shortage, something that occurs rather often in changing environments. Though we expected the colony to forage to its maximum capacity, the flow of food entering the colony is finely tuned to the colony's needs. Indeed the food-flow decreases proportionally to the food that has already been harvested, liberating the work-force for other tasks.

## Introduction

Spatial information plays an essential role in the dynamics of biological or artificial systems. Many studies have been dedicated to network dynamics [1] and the patterns of diffusion in ecological systems [2] or in unicellular populations [3]. In contrast, the dynamics and spatial organisation of highly integrated societies, such as social insects, are poorly understood. The spatial components of a society define its' social stucture, and vice versa, via a network of feedback loops.

Incorporating division of labour and complex communication pathways, communities of social insects are a prime example of social organisation. For example, foragers have to harvest, often cooperatively, the food requirements of the whole nest. A chain of demand, principally derived from the larvae and the queen, regulates foraging activity according to the colony's needs [4], [5]. The spatial distribution of individuals in the colony has long been overlooked, despite the fact that it clearly affects social activity, communication, and various regulation pathways. Intuitively, the activity of the colony also affects their spatial organization.

Several studies have demonstrated a relationship between task specialization and position inside the nest [6], [7] which, according to some authors, might be the consequence of a response to a gradient or heterogeneity [8]–[10]. Nevertheless, we cannot ignore the aggregation phenomenon [11] and the organisation occurring within and between aggregates. This aggregation process can also be observed for resources: e.g. combs inside a hive are organized so that pollen cells (the principal food source for the larvae) are at the periphery of the brood combs whereas honey combs are external [7], [12]. This spatial aggregation of food reserves is naturally the result of worker ant behaviour.

The direct exchange of food (as opposed to separate feeding from a common food source) is widespread among animal species from unicellular organisms to mammals, and is often considered to be a means of information exchange e.g. in presocial insects [13], [14], in spiders [15], in vampire bats [16] and birds [17]. However, in animal societies, the importance of food exchanges has reached its pinnacle in the colonial organisms and in social insects [18]–[20].

Food exchanges are at the basis of social organisation and fundamental to the division of labour in societies. For social insect species that rely principally on liquid food, foragers ingest food at the source, store it in their ‘social stomach’ and bring it back to the nest [21], [22]. The weight that can be stored in the social stomach is comparable to the weight of the ant itself [23]. Once in the midgut, food can be either digested or regurgitated to another worker by trophallaxis; defined as the transfer of fluids among members of a society through mouth-to-mouth (stomodeal) or anus-to-mouth (proctodeal) feeding [24]. Because of its importance in social organization, some authors support the hypothesis that trophallaxis is one of the key factors necessary for the evolution of eusociality [25]–[29]. Beyond its main feeding role, food exchanges reinforce social cohesion and trophallaxis is a communication channel [30], [31]. According to the gestalt theory [32], trophallaxis facilitates rapid homogenization of the cuticlar hydrocarbons that are the basis of colonial odour [33], [34]. Various pheromones may also be exchanged during trophallaxis [35], [36]. In termites and cockroaches, proctodeal trophallaxis is crucial for replacing the gut endosymbionts that are lost at every molt [13].

Despite the central role of trophallaxis, food-flow and spatial organization of food reserves within a colony, these subjects have been little studied. To date, most work in this area has focussed on the flow of food in fire ants *Solenopsis invicta* in relation to its possible contribution to pest control. These studies mainly focussed on the final distribution of food in the nest [37]–[42], on the global distribution of food [43], [44] and on the quantities received by different groups [45], [46]. These studies used dyes [47], [48] or radioactive markers [40], [41], [43], [46], [49] to track food, but the measures of flow (i.e. the quantity of food received as a function of time) were made on a limited group of individuals removed from the colony. These methodologies are invasive and do not yield information on the dynamics of the spatial distribution of the food. The lack of spatio-temporal studies is mainly due to the absence of a methodology that allows accurate monitoring of food-flow.

We present here, to our knowledge for the first time, a spatio-temporal study of the distribution of food in ant nests. Using a novel approach based on medical imagery (scintigraphy), we investigate the global dynamics and kinetics of food-flow in a spatial framework. We evaluate some of the quantitative rules which affect food-flow, such as how the harvested quantity regulates food-flow in the nest or how food aggregation evolves in *Formica fusca* nests.

## Methods

### 1. Biological model

*Formica fusca* L. is a widespread species in palearctic regions, present in a large number of habitats e.g. meadows, forests and urban areas. Colonies number between 500 and 2000 workers, and are weakly polygynous [50]. Workers measure from 4.5 to 7.5 mm, castes are temporal (age-based). *Formica fusca* has an opportunistic diet and exploits honeydew produced by aphids [51], [52]. As a polydomous species, the colony occupies at least two spatially separated nests [53]. Our set-up could be seen as a “model” of such nest. The experimental constraints force us to work in certain conditions (small nests) we have therefore chosen to work with a polydomous species.

### 2. Collections

Four mother colonies were harvested in Ermenonville (France) in August 2006. After two months of hibernation at 5°C, we formed nine groups each of 200 randomly-chosen workers (two groups from each of three first mother colonies and three groups from the fourth colony). We choose at first to avoid brood and study the food-flow in the “simplest” context.

Each group is, henceforth, referred to as a colony. Colonies were housed in plexiglass nests. The nest area was covered with a red filter (Rosco color filter, e-colour #19: Fire). The inner surface of the nest measured 10×10 cm and had a height of 2 mm. This height was sufficient for the workers to move freely, while hindering the formation of multi-layered aggregates. A thin gypsum layer cast at one end of the nest was watered daily to keep the nest moist. The gallery leading to the food source measured 3×20 cm, and its inner walls (3 cm high) were coated with Fluon® to prevent foragers from escaping. Colonies were maintained in the laboratory at 23±2°C, with a twelve hour photoperiod. Food supplies were *ad libitum* 1 M sucrose solution and a half mealworm (about 100 mg, *Tenebrio molitor*) twice a week. Six days prior to the experiment, food was removed [54], so the ants only had access to water.

### 3. Food Source and scintigraphy

In order to investigate workers' spatial organisation, we filmed each experiment with a webcam (1 picture/2 seconds). This allowed us to record the location of returning foragers.

To monitor the food-flow in the nest, we used a medical imagery technique, scintigraphy, which permits the monitoring of radioactive elements. A radioactive marker (technetium-99m) was mixed to a 0.5 M sucrose solution supplied to the ants. Technetium-99m is a by-product of the fission of Uranium-235 and has a short half-life (6 h 02) and rapidly eliminated (within 3 days in humans[55], [56]).The tracer emits low-energy gamma photons that are recorded by a gamma camera.

Scintigraphy measures the radioactivity within each surface element (pixel) according to its spatial coordinates (*x*, *y*) over time. One pixel corresponds to an area of 4.5 mm ×4.5 mm. The nest (10×10 cm) is covered by 494 pixels. The distribution of signal intensity across the pixels corresponds to the distribution of food within the nest at each time point of the experiment. The intensity is mesured by the number of counts (number of gamma photons recorded) for an interval of time (in our case, 30 seconds). A concentration of 3 mCi technetium-99m was used, based on prior studies using radioactivity [42], [45] and the sensitivity of the gamma camera. Monitoring of radioactivity within the nest commenced when the labelled sucrose was placed in the experimental setup and continued for a period of three consecutive hours. Technetium99-m proved to be an appropriate tracer: its half-life is sufficiently short to allow repeated experiments within a short time frame, and sufficiently long for the full dynamics of the food distribution within the nest to be monitored.

The volume of labelled sucrose used was 1 ml since, in test experiments, we observed that this quantity was never completely consumed during the experimental period. At least four nests were monitored simultaneously and each colony was tested only once a week. Six colonies were experimented upon twice and three were used only once overall.

### 4. Quantification of the radioactive intensity

#### Treatment of the data obtained by scintigraphy

The radioactive decay between the beginning and end of a 30-second exposure was negligible; the initial radioactive intensity diminishes by only 1‰. Therefore, the quantity of emitted photons during a single exposure can be considered constant. Over the course of a three-hour experiment, however, the radioactive decay is not negligible. Therefore, we used the radioactive decay equation to correct the collected data. The fitting of the food-flow was based on time intervals of 4 minutes.

#### Quantification of the radioactive intensity

In order to interpret a given radioactive intensity as a volume of sucrose solution, we made two types of measurement. First, we measured the radioactive signal of a forager returning to the nest. By overlapping the pictures obtained with the webcam and the ones obtained by scintigraphy, we could localize a forager returning to the nest and determine its radioactive signal. We carried out these individual measurements for 33 workers chosen randomly from all replicates. In a second step, we measured the volumes ingested by foragers using the method described by Mailleux et al. [23]. We approximated the ingested volume (*V_ingested_*) by subtracting the abdomen volume before ingestion (*V*) from that after ingestion (*V′*), assuming the abdomen to be an ellipsoid of revolution (equation 1): 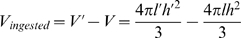
(1) where *l* (*l′*) is half the length of the abdomen and *h* (*h′*) is half the height of the abdomen before (and after) ingestion. Volume is measured in microlitres, and distance measurements (*l*, *h*) in millimeters.

#### Volume and radioactivity

For *Lasius niger*, Mailleux et al. [23] have shown that the fraction of ants (*F*) that have come in contact with a food source and that continue to drink after having ingested a volume *V* is given by the following equation: 
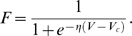
(2)
*V_c_*, the desired volume, is the threshold value and is close to the average volume ingested by workers. *η* is a parameter modulating the drinking behaviour.

#### Spatial repartition of the food

To have a better insight of the process of food sharing, we focussed on the spatial distribution of the radioactive intensities and its evolution during the experiment. We chose to study three complementary parameters (inspired by [10]):

1. Centre of gravity: we measured the centre of gravity of the radiocative surface for each exposure and calculated the distance between the centre of gravity of two successive pictures (two minute intervals). This gave a measure of the spatial stability of the food inside the nest. We also determined the intensity of pixels as a function of the distance from the centre of gravity.
2. 25-pixel square: for each exposure (two minute intervals) a square of 5×5 pixels was positioned so that it covered the zone where radioactivity is maximal.
3. Minimum surface representing 50% of the radioactivity: for each exposure (2 min intervals) we defined the smallest zone of contiguous pixels containing at least 50% of the total radioactivity present in the nest.

#### Radioactive contamination of the substrate

We did not manipulate the ants during our series of experiments. Therefore, we were not able to directly measure the radioactive background of the empty nest after the experiment. We carried out separate measurements in order to confirm that contamination of the substrate was negligible. The number of pixels recorded as radioactive at minute 15 represents only 10±7% (mean±SD, *n* = 15) of the total number of pixels contaminated since the start of an experiment, indicating that the pixels that a radioactive ant moves across do not retain a detectable radioactive signal.

## Results

### 1. Imagery

**Figure 1:**
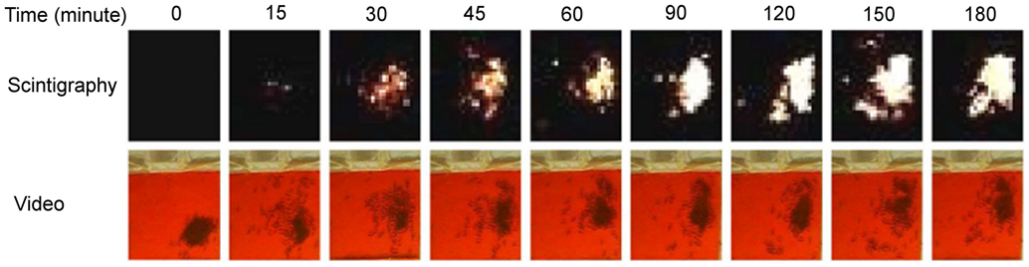
Images of the radioactivity and ants as food enters the nest. Example of pictures obtained during the three hours of observation. First row: pictures obtained by scintigraphy, second row; pictures obtained with the webcam. Nest entrance located at the bottom.

Figure 1 illustrates the typical evolution of an experiment: the top row shows the pictures obtained from scintigraphy monitoring, and the lower row shows the matching webcam pictures. As seen in the scintigraphy pictures, radioactivity spreads very quickly within the colony. The radioactive signal is weak until half an hour after the beginning of the experiment and grows in intensity thereafter. The webcam pictures show a stable spatial distribution of the individuals: the aggregate remains rather compact during the whole experiment even though (in this case) its position changes.

### 2. Volume and radioactivity

The experimental volumes that foragers carry, measured with scintigraphy and with ellipsoid approximation, conform to the theorical distribution of ingested volumes (equation (2) least square fitting, see Table 1). Both measurements give similar values for *η* and *V_c_*. The ratio between the two *critical volumes* (*V_c_*) obtained and the ratio between the two *mean volumes* (*V_m_*) is similar for both methods (1.92 and 1.89, respectively). This ratio gives us the intensity of the radioactive signal for 1 mm^3^ of ingested solution.

**Table 1:** Parameters obtained of the volumes ingested by foragers.

Conditions :	*η*	*V_c_*	*V_m_±SD*	*r^2^*	*SD*	*F*	number of replicates
Radioactivity (2 sec intervals)	0.31	12.7	15.0±7.6	0.97	0.32	*F*_1,32_ = 615.5	33
Volume (mm^3^)	0.35	6.7	7.8±5.4	0.97	0.20	*F*_1,24_ = 362.6	25

Parameters of the probability of ingesting food obtained with the two methods radioactivity and volume measurements. *η* is the parameter modulating the drinking behaviour, *V_c_* the critical volume and *V_m_* the mean ingested volume (least square fitting).

### 3. Dynamics of food-flow

#### Evolution of the radioactive intensity

**Figure 2:**
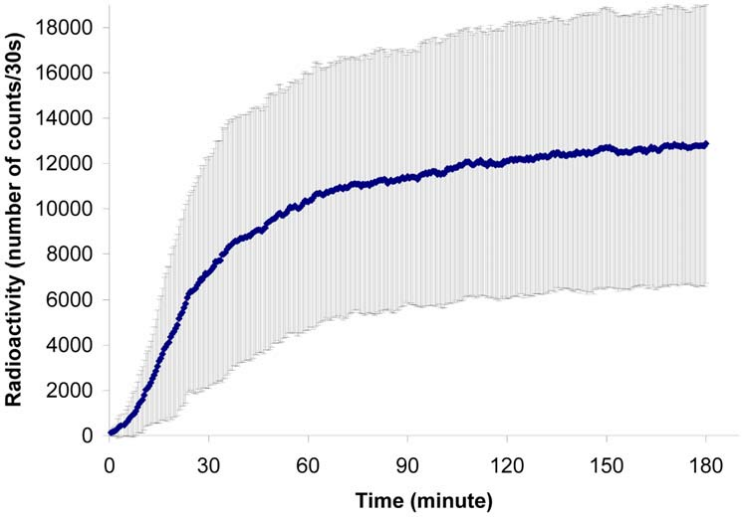
Entering food flow. Evolution of the average radioactivity for the three hours of experiment (number of counts per 30 seconds). Mean values (♦), standard deviation (bars) (n = 15).

All experiments, including the average curve, showed the same trend (fig. 2): some experiments were characterized by a shorter/longer delay before the radioactivity rapidly increased; after one hour, an average of 80%±9% (m±SD, *n* = 15) of the total amount of foraged radioactive labelled sucrose solution had entered the nest. All experiments presented a well-defined plateau (one-way ANOVA with the simple contrast method *F*_14,1470_ = 31.3 for time >114.5; *p*>0.05). The plateau values are normally distributed (Kolmogorov-Smirnov test, *D* = 0.09, *p*>0.10, *n* = 15).

We used the radioactive signal intensity to quantify food-flow. The total average quantity brought back to the nest equals 446±226 mm^3^ (*n* = 15). The total harvested quantities are normally distributed (Kolmogorov-Smirnov test, *D* = 0.09, *p*>0.10, *n* = 15).

Considering the average load per forager (see Table 1) 7.80±5.40 mm^3^, a total of approximatively 57 journeys accounts for the total quantity of food present in the nest at the end of the experiment. This is less than the capacity of the colony, if we consider that all workers can hold as much food as a forager.

#### Modelling of the food-flow

**Figure 3:**
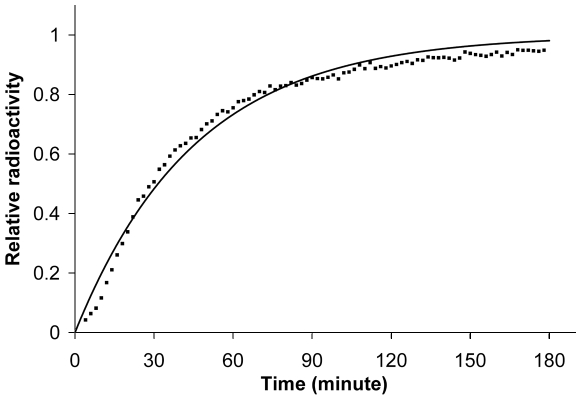
Fitting of the food flow. Evolution of the radioactive intensity during the three hours of observation. Normalized theoretical values (▪), normalized mean experimental values (n = 15) (♦). Standard deviation see Figure 2.

Our results suggest that the quantity of harvested food at time *t* acts as a negative feedback on the food-flow entering the colony, per unit time. The experimental curves (fig. 2 and 3) show that the harvested quantity reaches a plateau value, which we refer to as the “colony-desired harvested volume” (*K*).

Let *Ф(t)* be the entering food-flow. The quantity of harvested food as a function of time can be written: 
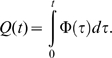
(3)


If *Q*(*t*) acts as negative feedback on the entering food-flow, we can relate the flow to the harvested quantity as follows: 
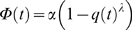
(4) with 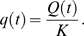
(5)
*q(t)* is the harvested quantity expressed as a fraction of the desired volume, *K* being the carrying capacity of the colony and corresponding to the total harvested food. *α* corresponds to the maximal food-flow.

The entering food-flow is proportional to the number of foragers and to the quantity that can be ingested by a forager, while the negative feedback acts on the number of active foragers [4], [57]. When *λ* is large, the regulation of the entering food follows an all-or-nothing rule, *i.e*. food enters the nest at a constant (maximal) rate until reserves have been met, at which point it ceases. Conversely, when *λ = *1, regulation is directly proportional to the quantity of food in the nest. Fitting of the food-flow with equation (4) gives us *λ* = 1.05 (95% confidence bounds: 0.60 and 1.50, *r* ^2^ = 0.89, *F*_14,74_ = 598.7, *SD* = 0.89) and *α* = 0.027 (95% confidence bounds: 0.0207 and 0.03255).

Taking *λ = *1, it follows from equations (4) and (5) that 
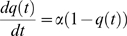
(6)


Equation (6) states that the food-flow depends on the total quantity of food that has been ingested or stored by the colony (*K*) and on the quantity of food already present in the colony (*q*).

Following integration of (6), we obtain: 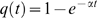
(7)


Thus, the quantity of food present inside the nest (*q*) follows and inverse exponential function, which implies that the food-flow (*dq/dt*) follows an exponential function (fig. 3). All experimental curves can be approximated by equation (7) (*r^2^*>0.80 for all 15 experiments) There is no correlation between the final signal intensity and the value of α (Spearman *r* = 0.42, *p* = 0.09, *n* = 15).

### 4. Dynamics of the contaminated surface

**Figure 4:**
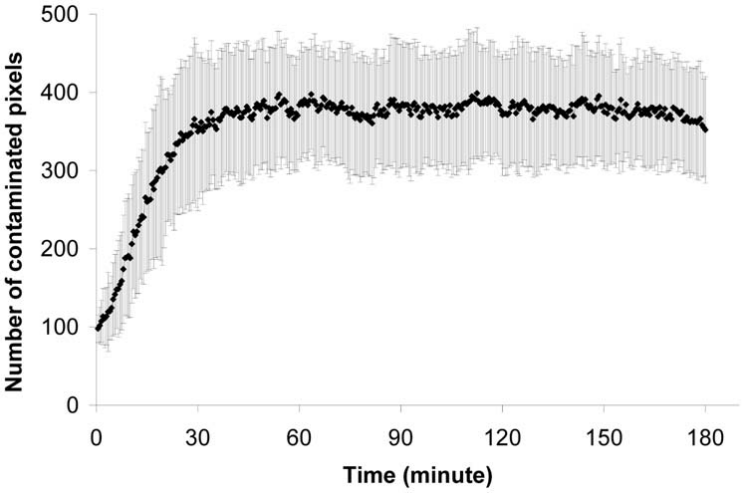
Contaminated surface. Evolution of the quantity of activated pixels during the trhee hours of experiment (n = 15). Mean values(♦) and standard deviation (bars).

We observe a rapid increase in the number of radioactive pixels. Within 25 minutes of initiating the experiment, the plateau value is reached (one-way ANOVA with the simple contrast method *F*_14,1335_ = 2.49 for *t*>25 minutes, *p*>0.05) which suggests that all contaminable pixels and, therefore ants, are radioactive (fig. 4). The mean number of contaminated pixels is 377.3±72.5, equal to an area of 7634 mm^2^. This area could be covered by an aggregate of 200 ants, according to the aggregate-surface fit defined by Depickère et al [11]: 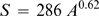
(8) where *S* is the surface of an aggregate in mm^2^ and *A*, the number of ants. The constant value of 286 was calculated on the basis of the value found for *L. niger* (*i.e.* 204) by Depickère et al. [11] and corrected by a factor of 1.4, which corresponds to the difference in size between *L. niger* and our biological model, *F. fusca*.

The number of contaminated pixels reaches its plateau value (*t* = 25 minutes, fig. 4) before the flow of food stops (*t* = 115 minutes, fig. 3). At the end of the experiment, 69.6±13.8% (m±SD, *n* = 15) of the total number of pixels covering the whole nest were activated. Because the surface covered by the radioactivity equals the surface occupied by an aggregate of 200 ants, we can surmise that ants rapidly receive radioactive food as it enters the nest. Subsequently, as more food enters the nest the mean intensity of contaminated pixels increases, indicating that food stocks are being accumulated.

**Figure 5:**
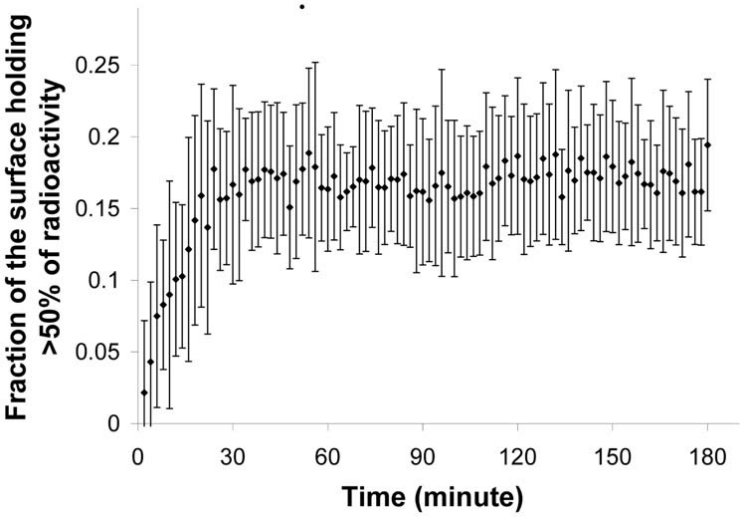
Food concentration: fraction of the surface holding 50% of the radioactivity. Evolution (minutes) of the relative average surface holding minimum 50% of the total radioactivity (n = 15). Mean values(♦) and standard deviation (bars).

The distribution of pixel intensity provides an initial insight into food sharing among ants. This distribution can be approximated by an exponential rule *P*(*I_p_*)≈exp(−*β*⋅*I_p_*) (for all 15 experiments: at *t* = 30 minutes, 60 minutes and 180 minutes, least square fitting, *r^2^*>0.9, *p*<0.001, *F_15,184_*>110,4, *SD*<1.09), where *β* is the inverse of the mean intensity per pixel and remains constant (≈0.03) up to 30 minutes after the start of an experiment. This distribution suggests high variability in pixel intensities and, therefore, in worker loadings; *i.e*. a small number of pixels (25%∼50 ants) represent a high proportion (80%) of the radioactivity. The surface of contiguous pixels holding minimum 50% of the radioactivity reaches a plateau value after 30 min (fig. 5) and represented an average of 17±7% of the contaminated pixels.

### 5. Spatial distribution of the food

#### Concentration of the food

The relative radioactivity contained in the 25-pixel square also rapidly reaches its plateau value after 30 minutes of experimentation (one-way ANOVA with the simple contrast method *F*_14,1335_ = 1.25, *p*>0.10). At the end of the three consecutive experimental hours, the square contains a total of 26.7±7% (*m±SD*, *n* = 15) of the radioactivity present inside the nest, whereas it only covers 7.3±1.3% of the contaminated surface. The results obtained from both indices (*i.e.* the minimum surface with 50% of the radioactivity and the 25-pixel square) are consistent with each other.

**Figure 6:**
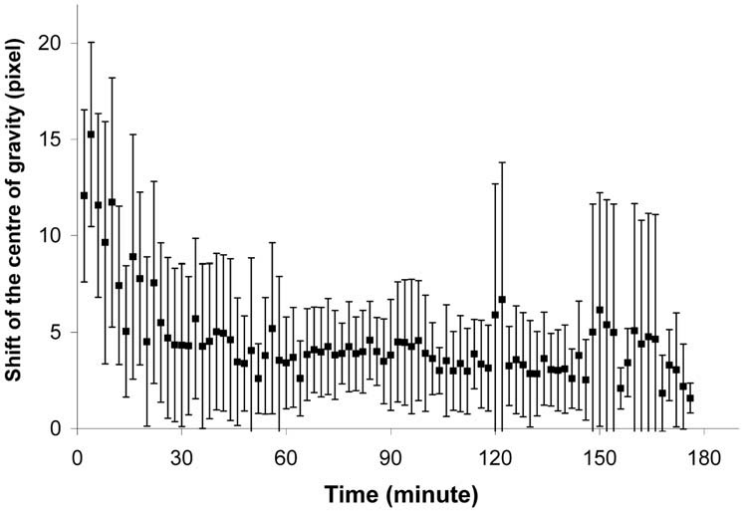
Spatial stability: shift of the centre of gravity. Displacement (in pixels) of the centre of gravity as a function of the time (minute, n = 15). Mean value (♦) and standard deviation (bars).

The mean distance between two centres of successive pictures rapidly decreases (fig. 6). After 25 minutes, the distance between the centre of two successive pictures is smaller than the mean distance between two random points of a square corresponding to the nest area (after 25 minutes, one sample *t* test, *t*_75_ = 68, *p*<0.0001, *n* = 77). We can therefore assert that the position of the zone of highest radioactive concentration is stable and that this stability arises quite early in the experiment.

#### Centralisation of the food

**Figure 7:**
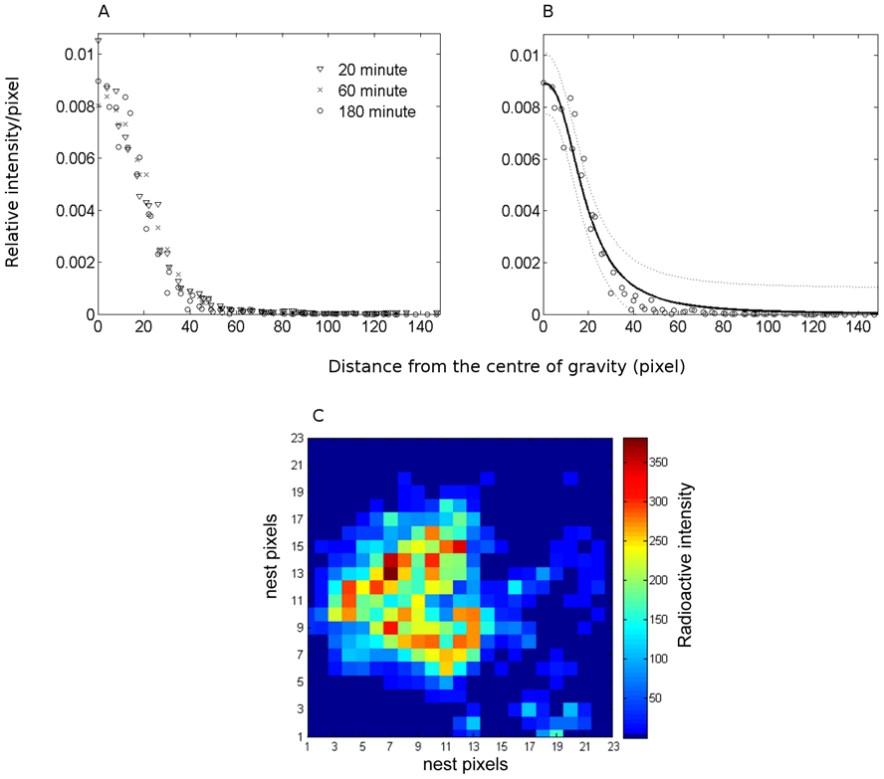
Central concentration of the food: Spatial distribution of radiolabelled sucrose. A. Distribution of the radioactive density per pixel as a function of the distance in pixel from the centre of gravity at 20 minutes (▾), 60 minutes (×) and 180 minutes (○). B. Experimental values at 180 minutes (○) and fit (equation 9). C. Example of the two-dimensional distribution of the radiolabelled sugar in a 23×23 pixels nest three hours into the experiment, number of counts per pixel summed during 30 seconds. The nest entrance is located at the bottom

We calculated the radioactive intensity as a function of the distance to the centre of gravity. The distribution of the radioactive density per pixel fits the following equation, whether or not the flow of food entering the colony ceased (fig. 7a): 
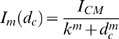
(9)


Where *I_CM_* is the relative intensity of the centre of gravity, *d_c_* is the distance to the centre of gravity, and *k* is the distance at which the radioctivity equals half the radioactivity of the centre of gravity. For *m*>2, there is a marked decrease of radioactivity as we move away from the centre of gravity whereas, with smaller values of *m*, the plateau zone where radioactivity equals that of the centre of gravity is longer (fig. 7a, b).

The evolution of the distribution shows a concentration and a centralisation of the food inside the nest (fig. 7c). This distribution follows the same principle throughout. The exponent is smaller (*m*≈2.5) at the beginning of the experiment which means that, as food enters the nest, food gets more concentrated around the centre of gravity (*m*≈4 at the end of the experiment, table 2). During the course of the entire experiment, the density of the centre of gravity of the radioactive surface remains constant (calculated every 20 minutes: Kruskal-Wallis test, *H*_14_ = 4.45, *n* = 15, *p* = 0.81).

**Table 2:** Distribution of the pixels' density around the centre of gravity.

Time (min.)	*I_CM_*	*P*	*K*	*r^2^*	*SD*	*F*	number of measures	number of replicates
30	0.038	2.53	4.03	0.98	2.6·10^−5^	735.7	66	15
60	0.040	3.76	5.07	0.99	8.5·10^−6^	2102.7	66	15
180	0.043	3.97	5.07	0.99	8.4·10^−6^	1904.4	66	15

Fitting with equation (9) of the distribution of the radioactive density as a function of the distance from the centre of gravity of the contaminated surface (least square fitting test).

## Discussion

The non-invasive method used in this study allows us to obtain reliable dynamic data on food distribution within ant nests, without having to handle colonies during the whole experiment. It allows a precise determination of harvested quantities, something traffic flow or behavioural indices provide with a lower confidence [58]. The radioactive method accurately detects one microlitre of the glucose solution and is well suited for individual measures. The correspondence between radioactive intensity and ingested volume enables quantitative validation of the method. Individual mesures of ingested volume match the threshold hypothesis decribed by Mailleux et al. [59].

The harvested quantities show a nearly linear increase before reaching a plateau value. During this initial build-up phase that lasts for about one hour, after which 80% of the total harvested food has entered the nest, with 0.8 loaded foragers enter the nest (or 6.4 mm^3^) per minute. Considering the average load per forager (8 mm^3^), an average of 57 journeys is sufficient to attain colony satiety. If we assume the individual storage capacity is equal to the loading capacity of a forager, the total capacity of a colony of *F. fusca* approaches 1600 mm^3^; a value far greater than the actual harvested quantity. So, either the volume stocked by a worker is less than the volume transported by a forager, or only a fraction of the colony acts as storage. The results we have obtained are consistent with those presented in the literature, harvesting stops spontaneously after a couple of hours, even though the food source is not depleted [5], [21].

Our results show that the global kinetics of the food-flow entering the colony is proportional to the available storage volume, which decreases with the harvested quantity. The difference between the experimental and the theoretical curve is probably due to the effect of recruitment. This difference rapidly becomes negligible. The regulation of the food-flow can be explained by the regulation of the activity of the foragers. For a forager, the probability per unit time to perform trophallaxis is proportional to the number of receptive workers that remain or, in other words, the available volume. Since a forager has to unload its food before leaving the nest for another trip to the food source, the time between two foraging trips increases as the available volume decreases [57] until the forager ultimately remains in the nest.

The response of a colony to starvation is classically described as an all-or-nothing response: the stocks present in the colony must be below a critical value for mass recruitement to occur and the colony harvests at maximum capacity until a threshold volume is reached. Indeed, mass recruitment only happens after a sufficiently long starvation period [59]. The dynamics of the flow of food seem to contradict this model. According to our observations, food exploitation starts as soon as the reserve has decreased. The all-or-nothing exploitation phenomenon may be due to the coupling of a positive feedback [60] and a negative feedback due to the colony stocks. This hypothesis is supported by the observations of Mailleux et al. [59]: the recruiting behaviour of foragers does not depend on the level of starvation of the colony; the negative feedback only arises from the difficulty for a forager to find a receiver. When foragers discover a food source and the available volume is small, the amplifying mechanisms that characterize recruitment dynamics do not work. In our experiments, due to the proximity between nest and food source, foragers easily and independently discover the food source. Therefore, food recruitment does not play a key role and food-flow is thus mainly controlled by the negative feedback. Similar negative feedbacks are at work in honey bee colonies [12], [61].

The contaminated surface varies very little between replicates. The number of contaminated pixels rapidly reaches its plateau value (at about 30 minutes), which matches the surface occupied by 200 ants [11]. Thus, all individuals quickly receive food. Our results are consistent with previous studies showing that the majority of workers receive food within the first hour of foraging [40], [49].

The maximum contaminated surface is reached twice as fast as the harvested quantity reaches its plateau value. Nevertheless, from the start, sharing of food among workers is heterogeneous. The distribution of pixel intensities shows that the majority of the workers receive small amounts of food, while a small number of ants hold large amounts of food. In a more detailed spatial analysis, we observe that, on average, 17% of the contaminated surface contains more than 50% of the radioactivity. Moreover, the radioactivity steeply decreases when the distance from the centre of gravity increases, a tendency that strengthens as the food flows into the nest and the stocks become concentrated. Due to these indices of food sharing, we show that the patterns of spatial concentration quickly stabilize (after 60 minutes). From the first hour, the distribution of the food remains unchanged until the end of the experiment. There is no diffusion, but re-concentration of the food occurs after the first 30 minutes. So, even though our nests are rather homogenous in composition and do not contain larvae (an important cause of heterogenous distribution of reserves [62] and of workers [10], [63], [64]), stocks remain heterogeneously distributed throughout the whole experiment. This implies that foragers accumulate stocks as more food enters the nest.

Localization of the high food concentration zones also shows considerable stability. The position of the centre of gravity and high food density zone settles after 30 minutes of the experiment. During the first minutes of the experiment, slight changes in radioactivity have a significant impact on determining high-density areas, *e.g*. the entrance of a forager or a load transfer by trophallaxis might greatly affect the position of the high-density zone and/or centre of gravity. Nevertheless, the aggregation process seems to rapidly overcome the perturbation (*i.e*. food entry) and individuals rapidly re-organize to reach what seems to be a base activity level. Considering all possible factors that could influence the measured parameters, it is quite striking that shifts in the centre of gravity rapidly decrease and stabilize.

Many studies have dealt with inter-individual differences in storage capacities/levels of ants [40], [45], [49]. Experimental results demonstrate two complementary trends: homogeneous food distribution between workers [42] or accumulation of large quantities of food among specialized workers [40], [44]. Our results show that a small fraction of aggregated individuals accumulate most of the food.

An important question is the relation between the distribution among individuals and the heterogeneous spatial distribution of stocks, as reported here. Like many other insects [14], [65], [66], ants exhibit high aggregate-forming capacity. The individual probability of moving decreases with the number of neighbours and is the basis of such self-organized aggregation. Moreover, chemical marking of the substrate by workers attracts ants back to the original spot where they were aggregated, even after a perturbation [67]. Our hypothesis is that the stock aggregations and their spatial stability at three levels: whole radioactive aggregates, highly radioactive surfaces, and their centres of gravity, are due to the coupling between the gregarious behaviour of ants and individual levels of storage. We assume that loaded individuals will have a lower probability of moving and will, therefore, be at the centre of the aggregate. To validate this hypothesis, we need to be able to better differentiate the spatial distribution of individuals and stocks. In further experiments, we will address this question by applying a double radioactive-labelling method: one to monitor the food-flow, the other to monitor the workers.

The length of trophallaxis chains, the mobility of individuals and their specialization assures a rapid and effective distribution of resources within the nest and reduce, for example, the queueing delay [68]. It seems essential that we understand how these factors combine and how they allow the colony to respond quickly to its needs and, particularly, those of the brood [69] in a variable environment.

Theoretical studies might help to identify the link between mechanisms, spatial organization and the collective functioning.

## References

[1] Newman M, Barabási A-L, Watts DJ (2006). The structure and dynamics of networks.

[2] Rietkerk M, van de Koppel J (2008). Regular pattern formation in real ecosystems.. Trends Ecol Evol.

[3] Cohen I, Ron I, Ben-Jacob E (2000). From branching to nebula patterning during colonial development of the *Paenibacillus alvei* bacteria.. Physica A.

[4] Cassill DL, Stuy A, Buck RG (1998). Emergent properties of food distribution among fire ant larvae.. J Theor Biol.

[5] Dussutour A, Simpson SJ (2008). Carbohydrate regulation in relation to colony growth in ants.. J Exp Biol.

[6] Backen SJ, Sendova-Franks AB, Franks NR (2000). Testing the limits of social resilience in ant colonies.. Behav Ecol Sociobiol.

[7] Camazine S, Deneubourg J-L, Franks NR, Sneyd J, Theraulaz G (2001). Self-Organization in Biological Systems.

[8] Ceuster R (1977). Social homeostasis in colonies of *Formica polyctena* (Hymenoptera Formicidae): nestform and temperature preferences.. Proceedings, 8th International Congress IUSSI.

[9] Cox MD, Blanchard GB (2000). Gaseous templates in ant nests.. J Theor Biol.

[10] Tschinkel WR (2004). The nest architecture of the Florida harvester ant, *Pogonomyrmex badius*.. J Insect Sci.

[11] Depickère S, Fresneau D, Deneubourg J-L (2004). A basis for spatial and social patterns in ant species: dynamics and mechanisms of aggregation.. J Insect Behav.

[12] Seeley TD (1995). The wisdom of the hive: The social physiology of honey bee colonies..

[13] Bell WJ, Roth LM, Nalepa CA (2007). Cockroaches.. Ecology, Behavior, and Natural History.

[14] Costa JT (2006). The Other Insect Societies.

[15] Gundermann J-L, Horel A, Roland C (1991). Mother-offspring food transfer in *Coelotes terrestris* (Araneae, Agelenidae).. J Arachnol.

[16] Wilkinson GS (1984). Reciprocal food sharing in the vampire bat.. Nature.

[17] Henderson BA (1975). Role of the chick's begging behavior in the regulation of parental feeding behavior of *Larus glaucescens*.. Condor.

[18] Dunn CW (2005). Complex colony-level organization of the deep-sea siphonophore *Bargmannia elongata* (Cnidaria, Hydrozoa) is directionally asymmetric and arises by the subdivision of pro-buds.. Dev Dynam.

[19] Wilson EO (1975). Sociobiology: the new synthesis..

[20] Mackie GO (1986). From aggregates to integrates: physiological aspects of modularity in colonial animals.. Phil Trans Roy Soc Lond B.

[21] Hölldobler B, Wilson EO (1990). The Ants.

[22] Wheeler WM (1918). A study of some ant larvae with a consideration of the origin and meaning of social habits among insects.. Proc Am Philos Soc.

[23] Mailleux AC, Deneubourg J-L, Detrain C (2000). How do ants assess food volume?. Anim Behav.

[24] Wheeler WM (1910). Ants: Their structure, development and behaviour..

[25] Aron S, Keller L, Passera L (2001). Role of resource availability on sex, caste and reproductive allocation ratios in the Argentine ant *Linepithema humile*.. J Anim Ecol.

[26] Hunt JH, Breed MD, Michener CD, Evans HE (1982). Trophallaxis and the evolution of eusocial Hymenoptera..

[27] McGlynn TP, Owen JP (2002). Food supplementation alters caste allocation in a natural population of *Pheidole flavens*, a dimorphic leaf-litter dwelling ant.. Insectes Soc.

[28] Wcislo WT, Gonzalez VH (2006). Social and ecological contexts of trophallaxis in facultatively social sweat bees, *Megalopta genalis* and *M. ecuadoria* (Hymenoptera, Halictidae).. Insectes Soc.

[29] Wilson EO (1971). The insect societies.

[30] Camazine S, Crailsheim K, Hrassnigg N, Robinson G, Leonhard B (1998). Protein trophallaxis and the regulation of pollen foraging by honey bees (*Apis mellifera* L.). Apidologie.

[31] Farina WM (1996). Food-exchange by foragers in the hive -a means of communication among honey bees ?. Behav Ecol Sociobiol.

[32] Crozier R, Dix M (1979). Analysis of two genetic models for innate components of colony odor in social Hymenoptera.. Behav Ecol Sociobiol.

[33] Dahbi A, Hefetz A, Cerdá X, Lenoir A (1999). Trophallaxis mediates uniformity of colony odor in *Cataglyphis iberica* ants (Hymenoptera, Formicidae).. J Insect Behav.

[34] Lenoir A, Fresneau D, Errard C, Hefetz A, Detrain C, Deneubourg J, Pasteels J (1999). Individuality and colonial identity in ants: the emergence of the social representation concept.. Information Processing in social insects.

[35] Le Conte Y, Hefetz A (2008). Primer pheromones in social hymenoptera.. Annu Rev Entomol.

[36] Meer R, Preston C, Hefetz A (2008). Queen regulates biogenic amine level and nestmate recognition in workers of the fire ant, *Solenopsis invicta*.. Naturwissenschaften.

[37] DeGrandi-Hoffman G, Hagler J (2000). The flow of incoming nectar through a honey bee (*Apis mellifera* L.) colony as revealed by a protein marker.. Insectes Soc.

[38] Crailsheim K (1992). The flow of jelly within a honeybee colony.. J Comp Physiol B.

[39] Crailsheim K (1991). Interadult feeding of jelly in honeybee (*Apis mellifera* L.). J Comp Physiol B.

[40] Markin P (1970). Food dsitribution within laboratory colonies of the argentine ant *Tridomyrmex humilis* (Mayr).. Insectes Soc.

[41] Sorensen AA, Busch TM, Vinson SB (1985). Control of food influx by temporal subeastes in the fire ant, *Solenopsis invicta*.. Behav Ecol Sociobiol.

[42] Sorensen AA, Vinson SB (1981). Quantitative food distribution studies within laboratory colonies of the imported fire ant, *Solenopsis invicta* (Buren).. Insectes Soc.

[43] Howard DF, Tschinkel WR (1980). The Effect of Colony Size and Starvation on Food Flow in the Fire Ant, *Solenopsis invicta* (Hymenoptera: Formicidae).. Behav Ecol Sociobiol.

[44] Wilson EO, Eisner T (1957). Quantitative studies of liquid food transmission in ants.. Insectes Soc.

[45] Bonavita-Cougourdan A, Passera L (1978). Étude comparative au moyen d'or radio-actif de l'alimentation des larves d'ouvrières et des larves de reine chez la Fourmi *Plagiolepis pygmaea* Latr.. Insectes Soc.

[46] Sorensen AA, Mirenda JT, Vinson SB (1981). Food exchange and distribution by three functional worker groups of the imported fire ant *Solenopsis invicta* (Buren).. Insectes Soc.

[47] Cassill DL, Tschinkel WR (1996). A duration constant for worker-to-larva trophallaxis in fire ants.. Insectes Soc.

[48] Cassill DL, Tschinkel WR (1999). Regulation of diet in the fire ant, *Solenopsis invicta*.. J Insect Behav.

[49] Howard DF, Tschinkel WR (1981). The flow of food in colonies of the fire ant, *Solenopsis invicta*: a multifactorial approach.. Physiol Entomol.

[50] Hannonen M, Sundström L (2003). Reproductive sharing among queens in the ant *Formica fusca*.. Behav Ecol.

[51] Andersen MC (1991). An ant-aphid interaction: *Formica fusca* and *Aphthargelia symphoricarpi* on Mount St. Helens.. Am Midl Nat.

[52] Cook SC, Davidson DW (2006). Nutritional and functional biology of exudate-feeding ants.. Entomol Exp Appl.

[53] Debout G, Schatz B, Elias M, McKey D (2007). Polydomy in ants: what we know, what we think we know, and what remains to be done.. Biol J Linn Soc.

[54] Wallis DI (1962). Aggressive behaviour in the ant *Formica fusca*.. Anim Behav.

[55] Beasley TM, Palmer HE, Nelp WB (1966). Distribution and excretion of technetium in humans.. Health Phys.

[56] Shukla S, Manni G, Cipriani C (1977). Behaviour of the pertechnetate ion in humans.. J Chromatogr.

[57] Mailleux A, Deneubourg J, Detrain C (2003). Regulation of ants' foraging to ressource productivity.. P Roy Soc Lond B.

[58] Cameron EZ, Stafford KJ, Linklater WL, Veltman CJ (1999). Suckling behaviour does not measure milk intake in horses.. Anim Behav.

[59] Mailleux AC, Detrain C, Deneubourg J-L (2006). Starvation drives a threshold triggering communication.. J Exp Biol.

[60] Sumpter DJT, Beekman M (2003). From nonlinearity to optimality: pheromone trail foraging by ants.. Anim Behav.

[61] Biesmeijer J (2003). The occurrence and context of tremble dancing in free-foraging honey bees (*Apis mellifera*).. Behav Ecol Sociobiol.

[62] Camazine S (1991). Self-organizing pattern formation on the combs of honey bee colonies.. Behav Ecol Sociobiol.

[63] Sempo G, Depickère S, Detrain C (2006). How brood influences caste aggregation patterns in the dimorphic ant species *Pheidole pallidula*.. Insectes Soc.

[64] Sendova-Franks AB, Franks NR (1995). Social resilience in individual worker ants and its role in division of labour.. P Roy Soc Lond B.

[65] Amé JM, Rivault C, Deneubourg J-L (2004). Cockroach aggregation based on strain odour recognition.. Anim Behav.

[66] Jeanson R, Rivault C, Deneubourg J-L, Blanco S, Fournier R (2005). Self-organized aggregation in cockroaches.. Anim Behav.

[67] Depickère S, Fresneau D, Deneubourg J-L (2004). Dynamics of aggregation in *Lasius niger* (Formicidae): influence of polyethism.. Insectes Soc.

[68] Anderson C, Boomsma JJ, Bartholdi JJ (2002). Task partitioning in insect societies: bucket brigades.. Insectes Soc.

[69] Cassill DL (2003). Rules of supply and demand regulate recruitment to food in an ant society.. Behav Ecol Sociobiol.

